# Fasting triglycerides as a predictor of incident diabetes, insulin resistance and β-cell function in a Canadian First Nation

**DOI:** 10.1080/22423982.2017.1310444

**Published:** 2017-04-13

**Authors:** Natalie D. Riediger, Kirsten Clark, Virginia Lukianchuk, Joanne Roulette, Sharon Bruce

**Affiliations:** ^a^ Department of Community Health Sciences, Rady Faculty of Health Sciences, University of Manitoba, Winnipeg, Canada; ^b^ Manitoba First Nations Centre for Aboriginal Health Research, Rady Faculty of Health Sciences, University of Manitoba, Winnipeg, Canada; ^c^ Department of Human Nutritional Sciences, Faculty of Agricultural and Food Sciences, University of Manitoba, Winnipeg, Canada; ^d^ Northern Remote Family Medicine Residency, University of Manitoba, Winnipeg, Canada; ^e^ Sandy Bay First Nation Health Centre, Sandy Bay First Nation, Canada;

**Keywords:** First Nation, diabetes, triglycerides, insulin resistance, cohort study, obesity, hypertriglyceridaemic waist

## Abstract

**Background**: Diabetes prevalence is substantially higher among Canadian First Nations populations than the non-First Nation population. Fasting serum triglycerides have been found to be an important predictor of incident diabetes among non-indigenous populations. However, there is a great need to understand diabetes progression within specific ethnic groups, particularly First Nations populations.

**Objective**: The purpose of this study was to test for an association between fasting serum triglycerides and incident diabetes, changes in insulin resistance and changes in β-cell function in a Manitoba First Nation cohort.

**Methods**: Study data were from two diabetes screening studies in Sandy Bay First Nation in Manitoba, Canada, collected in 2002/2003 and 2011/2012. The cohort was composed of respondents to both screening studies (n=171). Fasting blood samples and anthropometric, health and demographic data were collected. A generalised linear model with Poisson distribution was used to test for an association between fasting triglycerides and incident diabetes.

**Results**: There were 35 incident cases of diabetes among 128 persons without diabetes at baseline. Participants who developed incident type 2 diabetes were significantly older and had significantly higher body mass index (BMI; p=0.012), total cholesterol (p=0.007), fasting triglycerides (p<0.001), and Homeostatic Model Assessment of Insulin Resistance (HOMA-IR) (p<0.001). Fasting triglyceride level was found to be a statistically significant positive predictor of incident diabetes independent of age, sex and waist circumference at baseline. Participants with triglycerides in the highest tertile (≥2.11 mmol/l) had a 4.0-times higher risk of developing incident diabetes compared to those in the lowest tertile (p=0.03). Notably, neither waist circumference nor BMI were significant predictors of incident diabetes independent of age, sex and triglycerides.

**Conclusion**: Fasting triglycerides may be useful as a clinical predictor of insulin resistance and diabetes development among First Nations populations. Unlike other ethnic groups, BMI and waist circumference may be less important factors in diabetes development.

## Introduction

Type 2 diabetes has reached epidemic status in both developed and developing nations globally [1]. Both incidence and prevalence rates are rapidly rising, with recent estimates projecting that by 2040, 642 million people worldwide will be living with type 2 diabetes [1]. According to the 2009–2010 Canadian Community Health Survey, 5.0% of the non-Indigenous population age 12 years and older has type 2 diabetes compared to 17.2% of First Nations adults (≥18 years old) on-reserve, as reported by the 2008–2010 Regional Health Survey [2]. The study community, Sandy Bay First Nation, also has a high documented incidence [3] and prevalence of diabetes and diabetes complications [4–6]; in 2011/2012, the age- and sex-standardised prevalence of type 2 diabetes was 39.4% among adults ≥18 years old [5]. As a chronic disease, the economic and psychosocial burden of diabetes is immense. The Canadian Diabetes Association estimates that the cost of diabetes in Canada is expected to rise to $16.9 billion in 2020 [7]. New prevention strategies and interventions are needed in order to decrease the burden of type 2 diabetes, particularly in high-risk groups such as First Nations populations.

An emerging risk factor for the development of type 2 diabetes is serum fasting triglycerides. Elevated fasting triglycerides are known to be present in type 2 diabetes. Several studies have suggested that elevated triglycerides are an independent risk factor for the future development of insulin resistance and diabetes [8–11], including among individuals who are insulin resistant alone and have not yet developed diabetes [12]. Of clinical significance, triglycerides have been found to be elevated more than 10 years before the onset of incident diabetes [13,14]. Identification of this risk factor may allow prediction of insulin resistance and diabetes years before clinical manifestations of disease [9]. Indeed, Olafsdottir and colleagues demonstrated that elevated triglycerides precede impaired glucose regulation and that utilisation of risk factors such as triglycerides may identify individuals at high risk of incident diabetes earlier than standard glucose screening [15]. As such, monitoring triglycerides in clinical practice may be useful in predicting the onset of incident diabetes. However, fasting triglycerides have not been extensively investigated as a predictor of incident diabetes in First Nations populations, with the exception of Sandy Lake First Nation [16], which is an Oji-Cree community. This study community is Ojibway. Given the known differences in diabetes progression among various ethnic groups [17,18], investigation among different First Nations populations is warranted.

The purpose of this study was to test for an association between fasting serum triglycerides and incident diabetes status, change in insulin resistance and change in β-cell function in a Manitoba First Nation cohort. The secondary objective was to test for an association between abdominal obesity and hypertriglyceridaemic waist, and incident diabetes, changes in insulin resistance and change in β-cell function in order to determine whether waist circumference adds to the explanatory value of fasting triglycerides. Elucidating early predictors of incident diabetes, like fasting triglycerides, would allow timely implementation of lifestyle and pharmacological interventions in order to reduce diabetes risk.

## Methods

### Design

The study community is Sandy Bay Ojibway First Nation, which is located approximately 200 km northwest of Winnipeg on the western shore of Lake Manitoba. As of July 2013, the on-reserve registered population was approximately 6000 people, with close to 50% under 19 years of age. Sandy Bay First Nation entered into a research partnership with Dr Sharon Bruce in 2002. The data utilised in this study were collected during two separate study periods. Baseline data (2002/2003) were collected from October 2002 to December 2003 [4]. Follow-up data (2011/2012) were collected between July 2011 and June 2012 [5]. Fasting blood work, anthropometric and questionnaire data were collected for both studies.

### Sample

All adults in the community of Sandy Bay aged 18 years or older, excluding those pregnant, were invited to participate during both study time points. Participants were required to be either a registered member of Sandy Bay Ojibway First Nation or a registered member of another First Nation community but residing in Sandy Bay. Study participants were recruited through local advertisements within the community and through door-to-door recruitment by community research assistants. A total of 482 participants were enrolled in the 2002/2003 study and 596 participants were included in the 2011/2012 study, of which 171 were returning participants. This report focuses on the 171 participants who participated in both the 2002/2003 and 2011/2012 studies, comprising an 8-year follow-up cohort. This 8-year follow-up cohort included men and women as well as those with and without diabetes.

### Measures

Data collection was performed in Sandy Bay First Nation and included fasting blood work, anthropometric and questionnaire data. Venous blood samples were taken by a registered nurse after a 12-hour fast, processed on site and then stored at −20°C. Blood samples were delivered to the Clinical Chemistry Laboratory at Health Sciences Centre in Winnipeg for measurement of clinical indicators. Total cholesterol (TC), high-density lipoprotein cholesterol (HDL-C) and triglycerides were measured using standard enzymatic methods. Low-density lipoprotein cholesterol (LDL-C) was calculated using the Friedewald equation [19]. Weight and height were assessed using a beam scale and a metric wall tape, respectively. Body mass index (BMI) was calculated as weight (kg)/height (m)^2^. Waist circumference was measured by identifying the natural waist with an inelastic tape [20]. Abdominal obesity is defined as ≥102 cm for men and ≥88 cm for women [21]. Hypertriglyceridaemic waist is defined as fasting triglycerides ≥2.0 mmol/l and waist circumference ≥90 cm for men and ≥85 cm for women [22].

Diabetes is defined as currently on an oral hypoglycaemic, self-declared or fasting glucose ≥7.0 mmol/l [23]. Insulin resistance (HOMA-IR) and β-cell function (HOMA-β-cell function) of each participant at baseline and at 8 years of follow-up were estimated using the Homeostatic Model Assessment. HOMA-IR was calculated using the formula (insulin (pmol/ml) × glucose (mmol/l/22.5). HOMA-β-cell was calculated using the formula (20 × insulin (pmol/ml)/(glucose (mmol/l) – 3.5) [24]. The changes in insulin resistance (ΔHOMA-IR) and β-cell function (ΔHOMA-β-cell) between follow-up and baseline were calculated by subtracting the value at baseline from that at follow-up. Therefore, a positive value denotes an increase in the respective measure over time and a negative value denotes a decrease in the respective measure over time.

### Analysis

Statistical analysis was completed using SPSS (version 22.0). Lipid measures (TC, LDL-C, HDL-C, TC/HDL-C ratio and triglycerides), fasting glucose and insulin, anthropometric characteristics (BMI and waist circumference) and hypertriglyceridaemic waist at baseline were compared between participants according to their diabetes status at follow-up, no diabetes, previously diagnosed diabetes and incident diabetes. The mean and standard deviation were reported for normally distributed continuous variables. The median and interquartile range were reported for non-normal distributions. Analyses of variance with Tukey tests were used to test for differences between the three groups. The Mann–Whitney U test was used to test for differences in fasting triglycerides between each of the three groups.

A generalised linear model with Poisson distribution was used to test for an association between fasting triglycerides and incident diabetes among participants without diabetes at baseline. Fasting triglycerides were divided into tertiles. Relative risks according to tertile of triglycerides were determined. Control variables included age, sex and BMI at baseline, as well as waist circumference at baseline (in a separate model). Sex interactions with fasting triglycerides were also tested. Abdominal obesity and hypertriglyceridaemic waist were also tested as a predictor of incident diabetes in separate models, adjusted for age at baseline and sex.

General linear models were used to investigate fasting triglycerides as a predictor of the change in insulin resistance (ΔHOMA-IR) and change in β-cell function (ΔHOMA-β-cell). Triglycerides were divided into tertiles. These models included all participants, including those with diabetes at baseline. Each model was adjusted for the outcome measure at baseline (i.e. HOMA-IR or HOMA-β-cell function at baseline). Additional variables adjusted for in the final model included age at baseline, sex and BMI at baseline, as well as waist circumference at baseline. Abdominal obesity and hypertriglyceridaemic waist were also tested as predictors of change in insulin resistance and change in β-cell function in separate models, adjusted for age, sex and the respective outcome measure at baseline. Sex interactions with fasting triglycerides were also tested.

### Ethics

This analysis and the aforementioned studies have been undertaken in partnership with the community of Sandy Bay First Nation using a community-based participatory research framework. Within this framework, members of the Sandy Bay First Nation play an active role in diabetes research within the community and work towards achieving better outcomes for those affected. A Community Diabetes Advisory Group approved this study. In addition, this study was approved by the University of Manitoba Health Research Ethics Board and each participant provided their individual informed consent.

## Results

A total of 171 participants were included in this study. The demographic characteristics of the sample at baseline (2002/2003) have been previously reported [3]. Among study participants, there was a high rate of unemployment and low educational attainment. Additionally, participants displayed a high prevalence of diabetes, smoking and obesity. Over the 8-year follow-up period, there were 35 incident cases of diabetes among 128 without diabetes at baseline.

The baseline characteristics of participants who developed incident diabetes were compared to those with pre-existing diabetes and those that did not develop diabetes (Table 1). Participants who developed incident type 2 diabetes were significantly older and had significantly higher BMI (p=0.012), total serum cholesterol (p=0.007), TC/HDL-C ratio (p=0.002), and serum triglycerides (p<0.001).

**Table 1.** Baseline characteristics of participants according to diabetes status.

Variable	No diabetes (n=93)	Previously diagnosed diabetes (n=43)	Incident diabetes (n=35)
Age (years)	32.2 ± 8.0	41.2 ± 9.2	37.7 ± 9.6
Sex			
Male	43 (45.7)	22 (52.4)	15 (42.9)
Female	50 (53.2)	21 (47.6)	20 (57.1)
BMI (kg/m^2^)	30.2 ± 7.0^a^	33.3 ± 6.2^b^	33.6 ± 5.4^b^
Waist circumference (cm)	100.2 ± 16.1^a^	112.4 ± 19.1^b^	106.2 ± 9.9^ab^
Total cholesterol (mmol/l)	4.5 ± 1.1^a^	5.2 ± 1.3^b^	5.1 ± 1.0^b^
LDL-C (mmol/l)	2.5 ± 0.9	2.7 ± 1.0	2.8 ± 0.8
HDL-C (mmol/l)	1.23 ± 0.31	1.09 ± 0.33	1.16 ± 0.34
TC/HDL-C	3.8 ± 1.3^a^	5.1 ± 1.7^b^	4.7 ± 1.6^b^
Fasting TG (mmol/l)	1.5 (1.0–1.8)^a^	2.3 (1.6–3.6)^b^	2.0 (1.4–3.0)^b^
Hypertriglyceridaemic waist	19 (20.9)^a^	25 (61.0)^b^	18 (54.5)^b^
Fasting glucose	5.0 (4.7–5.4)^a^	8.9 (7.3–12.8)^b^	5.50 (5.20–6.10)^c^
Fasting insulin	79.0 (40.0–129.0)^a^	132.0 (69.0–169.0)^b^	126.5 (96.0–187.5)^b^

Results presented as mean ± standard deviation, median (interquartile range) or n (%); ^ab^values without identical superscript letter within a row are significantly different (p<0.05) according to analysis of variance with Tukey tests, Mann–Whitney U tests or χ^2^ tests.

Fasting serum triglycerides was found to be a statistically significant positive predictor of incident diabetes independent of age, sex and BMI at baseline (Table 2; model 4), as well as independent of age, sex and waist circumference at baseline (model 5). After adjustment for age, sex and waist circumference at baseline, subjects with serum triglycerides in the highest tertile (≥2.11 mmol/l) had a 4.0-times greater risk of developing incident diabetes compared to those in the lowest tertile (p=0.041). Participants with triglyceride levels in the middle tertile did not have a significantly greater risk of developing incident diabetes compared to those in the reference group (p=0.109). Notably, BMI at baseline and waist circumference, as continuous variables, were not found to be significant predictors of incident diabetes independent of age, sex and triglycerides (p=0.152 and p=0.370, respectively). An interaction between sex and triglycerides was not statistically significant (highest tertile compared to reference group, p=0.875; middle tertile compared to reference group, p=0.689). Hypertriglyceridaemic waist is associated with a 2.3-times greater risk for incident diabetes, independent of age and sex (p=0.022) (Table 3). Abdominal obesity was not associated with a significantly increased risk of incident diabetes.

**Table 2.** Fasting triglycerides as a predictor of incident type 2 diabetes after an 8-year follow-up period.

Model	TG (mmol/l)	β (SE)	Relative risk	p-value
1	<1.36	Reference	Reference	–
	1.36–2.10	0.988 (0.521)	2.7	0.058
	>2.10	1.548 (0.512)	4.7	0.003
2	<1.36	Reference	Reference	–
	1.36–2.10	0.787 (0.533)	2.2	0.140
	>2.10	1.309 (0.529)	3.7	0.013
3	<1.36	Reference	Reference	–
	1.36–2.10	0.786 (0.533)	2.2	0.141
	>2.10	1.308 (0.529)	3.7	0.013
4	<1.36	Reference	Reference	–
	1.36–2.10	0.683 (0.537)	2.0	0.204
	>2.10	1.116 (0.538)	3.2	0.038
5	<1.36	Reference	Reference	–
	1.36–2.10	0.933 (0.581)	2.5	0.109
	>2.10	1.375 (0.581)	4.0	0.041

Model 1, unadjusted.

Model 2, adjusted for age at baseline.

Model 3, adjusted for age at baseline and sex.

Model 4, adjusted for age at baseline, sex and BMI at baseline.

Model 5, adjusted for age at baseline, sex and waist circumference.

**Table 3.** Comparison of fasting triglycerides, abdominal obesity and hypertriglyceridaemic waist as predictors of incident diabetes and change in insulin resistance.

Outcome	Predictor	β (SE)	Relative risk	p-value
Incident diabetes^a^	Triglycerides (mmol/l)			
	<1.36	Reference	Reference	-
	1.36–2.10	0.786 (0.533)	2.2	0.141
	>2.10	1.308 (0.529)	3.7	0.013
	Abdominal obesity			
	No	Reference	Reference	-
	Yes	0.821 (0.490)	2.3	0.094
	Hypertriglyceridaemic waist			
	No	Reference	Reference	-
	Yes	0.825 (0.362)	2.3	0.022
Change in insulin resistance^b^	Triglycerides (mmol/l)		n/a	0.005
	<1.36	Reference		0.012
	1.36–2.10	2.243 (0.801) 2.138 (0.854)		
	>2.10			
	Abdominal obesity		n/a	0.101
	No	Reference		
	Yes	1.395 (0.850)		
	Hypertriglyceridaemic waist		n/a	0.151
	No	Reference		
	Yes	1.047 (0.729)		

^a^ Generalised linear model with Poisson distribution, adjusted for age at baseline and sex.

^b^ General linear model, adjusted for HOMA-IR at baseline, age at baseline and sex.

Fasting serum triglycerides was found to be a statistically significant positive predictor of the change in HOMA-IR, independent of age, sex, HOMA-IR at baseline and waist circumference at baseline (Table 4, model 3); participants with serum triglycerides in both the middle and highest tertiles had a significantly greater increase in HOMA-IR over time compared to those in the lowest tertile (p=0.008 and p=0.011, respectively). Subjects with triglyceride levels in both the middle and highest tertiles demonstrated an increase in HOMA-IR of 2 units compared to the reference group. It should be noted that only three participants reported taking exogenous insulin treatment at follow-up. BMI and waist circumference at baseline were not significant predictors of the change in HOMA-IR, independent of age, sex, HOMA-IR at baseline and fasting triglycerides (p=0.406 and p=0.913, respectively). An interaction between sex and triglycerides was not significant. Abdominal obesity and hypertriglyceridaemic waist were not significant predictors of change in HOMA-IR independent of age, sex and HOMA-IR at baseline (Table 3).

**Table 4.** Fasting triglycerides as a predictor of change in HOMA-IR after an 8-year follow-up period.

Model	TG (mmol/l)	β (SE)	p-value
1	<1.36	Reference	–
	1.36–2.10	2.243 (0.801)	0.005
	>2.10	2.138 (0.854)	0.012
2	<1.36	Reference	–
	1.36–2.10	2.008 (0.810)	0.013
	>2.10	2.071 (0.872)	0.018
3	<1.36	Reference	–
	1.36–2.10	2.193 (0.823)	0.008
	>2.10	2.270 (0.890)	0.011

Model 1, adjusted for HOMA-IR at baseline, age at baseline and sex.

Model 2, adjusted for HOMA-IR at baseline, age at baseline, sex and BMI at baseline.

Model 3, adjusted for HOMA-IR at baseline, age at baseline, sex and waist circumference at baseline.

Regarding triglycerides as a predictor of the change in HOMA-β-cell function, statistical analysis did not converge due to the small sample size in combination with the number of control variables. Unadjusted results were not meaningful and are not reported here.

## Discussion

Overall, our findings indicated significantly higher BMI, triglycerides and insulin resistance at baseline among participants who developed incident diabetes compared to those who did not. Additionally, we found that fasting serum triglycerides was a significant, independent positive predictor of both incident diabetes and worsening insulin resistance in this First Nations population. Regarding incident diabetes, only those with serum triglycerides in the highest tertile (>2.11 mmol/l) at baseline were at a significantly greater risk of developing incident diabetes. In contrast, baseline triglycerides in both the middle (1.36–2.11 mmol/l) and highest tertiles predicted approximately the same increase in insulin resistance. BMI and waist circumference were not significant predictors of incident diabetes or change in insulin resistance in the fully adjusted model. Furthermore, triglycerides alone were a better predictor of both incident diabetes and change in HOMA-IR than hypertriglyceridaemic waist.

These results support the hypothesis that triglycerides are an independent risk factor for worsening insulin resistance and incident diabetes in this population. While many studies have demonstrated this in European populations [8–11], our study provides support for the same relationship among an Ojibway First Nation cohort. The only other reported results from a longitudinal study among a Canadian First Nations population (Sandy Lake First Nation), to our knowledge, also observed that fasting triglycerides was a significant predictor of incident diabetes [16]. These results are important because it is necessary to understand the development of diabetes among First Nations as a unique ethnic group. Acknowledgement of this relationship may allow for diabetes risk reduction to be implemented using medications that lower serum triglycerides. Fibrates are one such class of medication used to lower triglycerides. For instance, Zafrir and Jain suggest that by reducing triglycerides, fibrates may positively affect glucose regulation and improve insulin sensitivity [25]. Additionally, Flory and colleagues suggest that bezafibrate can help prevent type 2 diabetes, as well as slow the progression of insulin resistance [26]. In this regard, lowering triglycerides may be a pharmacological target in diabetes prevention, although further investigation into whether lowering triglycerides leads to reduced morbidity or mortality is needed.

Our study suggests that both the middle and highest tertiles of triglycerides have similar risks for worsening insulin resistance, unlike incident diabetes. In contrast to our results, Moro and colleagues demonstrated a dose–response relationship between serum triglycerides and insulin resistance [27]. These differences may be accounted for by interethnic variations in the relationship between lipid profiles and insulin resistance [28]. It is thus necessary to consider how ethnic differences between populations may affect the relationship between triglycerides and insulin resistance.

Interestingly, BMI and waist circumference were not significant predictors of incident diabetes or change in insulin resistance. Thus, our findings suggest that triglycerides are a better predictor of incident diabetes and worsening insulin resistance than BMI or waist circumference in this First Nations population. Furthermore, the addition of waist circumference using the hypertriglyceridaemic waist variable did not add to the prediction of either incident diabetes or worsening insulin resistance. This is contrary to some of the existing literature regarding measures of adiposity and diabetes. For instance, Hjellvik and colleagues demonstrated that BMI was the strongest predictor of type 2 diabetes in a Norwegian population [10]. Similarly, Koller and colleagues reported that obesity was a significant predictor of diabetes incidence among an Alaskan Native cohort. These differences may be explained by variations in body shape between ethnic groups. For example, First Nations populations display a high prevalence of abdominal obesity compared to other ethnicities [29]. Razak and colleagues have demonstrated ethnic variations in lipid and glucose measures among those belonging to the same BMI category related to the pattern of adipose tissue distribution [30]. Increasing abdominal obesity is associated with increased lipid and glucose measures among individuals of the same BMI. Thus, due to the predisposition of First Nations populations to develop abdominal obesity, it is possible that BMI differs in its predictability of incident diabetes and insulin resistance among First Nations groups. However, abdominal obesity was also not found to be a significant predictor of incident diabetes or change in insulin resistance in the present study. In this regard, Egeland and colleagues reported that hypertriglyceridaemic waist was a significantly associated with diabetes among an Inuit sample, whereas abdominal obesity was not [31], indicating that triglycerides are a critical component of diabetes prediction in that population. Perhaps as the prevalence/incidence of diabetes increases in a population, measures of adiposity become a less important predictor.

We did not note any sex differences in the relationship between triglycerides and incident diabetes or change in insulin resistance. Similarly, Ley and colleagues also reported in Sandy Lake First Nation that there were no significant sex interactions with individual lipid measures on diabetes incidence [24]. It is possible that the lack of sex difference in our study may be partially explained by the limited sample size and the power to detect any sex differences. Hopkins and colleagues have reported sex differences in the relationship between obesity/abdominal obesity and other metabolic risk factors among three combined Alaska Native study cohorts [32], such that the relationships are less strong among women. The lack of sex differences in the relationships may also be explained by the lack of sex differences in the prevalence and incidence of diabetes overall in the study sample as compared to the sex differences reported in other populations [32], as well as other First Nations populations [33–35].

Limitations of this study include the relatively small sample size collected from a single population and the use of fasting glucose to define diabetes rather than glucose tolerance tests. Important strengths include the longstanding relationship established with the study community and the 8-year follow-up period.

In conclusion, this study is particularly useful in that it provides longitudinal data among a First Nations population, an ethnic group that is at particularly high risk for diabetes with a disproportionate burden of disease. This analysis has identified fasting triglycerides as an independent positive predictor of incident diabetes, as well as change in insulin resistance, in a First Nations population. Such information may help to identify individuals who are at high risk of diabetes, as well as providing a new target for diabetes risk reduction that is superior to BMI or waist circumference.
